# Molecular and phenotypic distinction of the very recently evolved insular subspecies *Mus musculus helgolandicus* ZIMMERMANN, 1953

**DOI:** 10.1186/s12862-015-0439-5

**Published:** 2015-08-14

**Authors:** Hiba Babiker, Diethard Tautz

**Affiliations:** Max Planck Institute for Evolutionary Biology, August-Thienemann Str. 2, 24306 Plön, Germany

## Abstract

**Background:**

Populations and subspecies of the house mouse *Mus musculus* were able to invade new regions worldwide in the wake of human expansion. Here we investigate the origin and colonization history of the house mouse inhabiting the small island of Heligoland on the German Bight *- Mus musculus helgolandicus*. It was first described by Zimmermann in 1953, based on morphological descriptions which were considered to be a mosaic between the subspecies *M. m. domesticus* and *M. m. musculus*. Since mice on islands are excellent evolutionary model systems, we have focused here on a molecular characterization and an extended phenotype analysis.

**Results:**

The molecular data show that the mice from Heligoland are derived from *M. m. domesticus* based on mitochondrial D-loop sequences as well as on four nuclear diagnostic markers, including one each from the sex-chromosomes. STRUCTURE analysis based on 21 microsatellite markers assigns Heligoland mice to a distinct population and D-loop network analysis suggests that they are derived from a single colonization event. In spite of mice from the mainland arriving by ships, they are apparently genetically refractory against further immigration. Mutation frequencies in complete mitochondrial genome sequences date the colonization age to approximately 400 years ago. Complete genome sequences from three animals revealed a genomic admixture with *M. m. musculus* genomic regions with at least 6.5 % of the genome affected. Geometric morphometric analysis of mandible shapes including skull samples from two time points during the last century suggest specific adaptations to a more carnivorous diet.

**Conclusions:**

The molecular and morphological analyses confirm that *M. m. helgolandicus* consists of a distinct evolutionary lineage with specific adaptations. It shows a remarkable resilience against genetic mixture with mainland populations of *M. m. domesticus* despite major disturbances in the past century and a high ship traffic. The genomic admixture with *M. m. musculus* genetic material may have contributed to the genomic distinction of the Heligoland mice. In spite of its young age, *M. m. helgolandicus* may thus be considered as a true subspecies of *Mus*, whose evolution was triggered through fast divergence on a small island.

**Electronic supplementary material:**

The online version of this article (doi:10.1186/s12862-015-0439-5) contains supplementary material, which is available to authorized users.

## Background

The colonization patterns on islands have long been of major interest for studying evolutionary processes [1, 2]. Islands are considered natural laboratories of new adaptations due to the restricted scale, isolation, and sharp boundaries. Colonizations are usually accompanied by adaptive changes, but it is a long standing question whether fast adaptations are constrained by the initial paucity of variation caused by a genetic bottleneck of only few arriving individuals [2].

The house mouse *Mus musculus* L. is, apart of its role as a model organism for biomedical research, also ideal for evolutionary studies, due to its history of colonization of many new areas and islands [3–5]. It originated in Southern Asia up to a million years ago, spread throughout the world in several waves and diversified into at least three major subspecies, *M. m. castaneus*, *M. m. domesticus* and *M. m. musculus. M. m. musculus* colonized Central and Eastern Europe and Northern Asia, *M. m. castaneus* Southern Asia, and *M. m. domesticus* has been introduced to Western Europe, Africa, Americas and Australia by ships and trading traffic. Along with human traffic, the house mouse subspecies were able to invade also oceanic islands, including sub-Antarctic islands without human settlements [6, 7]. Phylogeographic studies based on house mouse mitochondrial genome (mtDNA) sequences link patterns of house mouse phylogeography and human historical movements, e.g. during the Iron Age and Viking Age [8, 9].

**Fig. 1:**
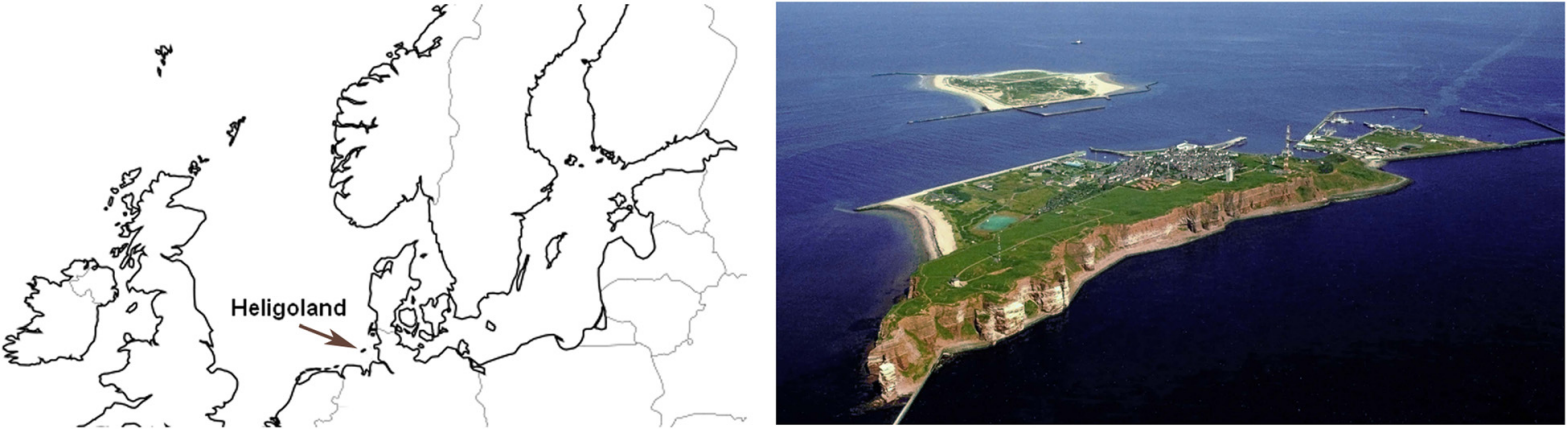
Location and aerial picture of Heligoland. Left: Map showing the geographical location of Heligoland Island in the North Sea next to North Western Germany (based on d-maps.com/carte.php?num_car = 2233&lang = en). Right: Aerial view from the West, showing the sandstone cliffs with the “Oberland”, the village in the “Unterland”, the harbor and the separate small island, the “Düne” (http://en.wikipedia.org/wiki/Heligoland). Both map and photo do not require copy right permissions

Here we focus on the house mouse inhabiting the Island of Heligoland. Heligoland (54° '11 N, 07° '53 E), is a small island in the North Sea in North Western Germany (Fig. 1) and consists nowadays of two small sub-islands. The main island which is known as Heligoland is a Triassic red sandstone rock, 1 km long, 61 m high and 46 km away from the German coast [10]. The smaller island, Dune Island, was attached to Heligoland until 1721, when a storm flood cut the connection. It is a sandy island with low sand dunes, lies now about 1 km east of Heligoland and harbors no house mouse population. The main island of Heligoland has two major distinctive land parts. The upper land is mostly surrounded by sandstone cliffs and the lower land is close to sea level and includes the island village as well as a harbor. Heligoland has had a turbulent history during the last century, including its use as a major naval military base during World War I and II, fierce bombing during the Second World War, evacuation of its population after the war and use as a bombing range by the British military until 1952. In 1947 it suffered one of the largest non-nuclear detonations in history, which resulted in a re-shaping of the topographic profile of the island. The population returned in 1952 and the island has since developed into a popular holiday resort with large numbers of visitors and many provisioning ships arriving throughout the year.

House mice on Heligoland were first mentioned in a vegetation and faunistic study from 1829 [11] and were described as a separate subspecies, *Mus musculus helgolandicus* by Zimmermann in 1953 [12]. This description was based on a unique combination of coloration, body size ratios and skull shape characters that are otherwise found in *M. m. domesticus* or *M. m. musculus*, i.e. the suggestion of a mosaic phenotype [12]. This description was revisited and confirmed in 1968 by Reichstein and Vauk [13]. However, *M. m. helgolandicus* remained underrepresented in later molecular genetic studies, with only a few samples and a restricted marker analysis [14–16].

In the present study we investigate the origin of *M. m. helgolandicus* from the two other subspecies inhabiting Europe, *M. m. domesticus* and *M. m. musculus*. We analyze the population structure using microsatellite markers, revisit their colonization history into the island using mtDNA sequences and estimate their age since the first colonization event based on the calculation of mutation frequencies obtained from whole mtDNA sequences. Using full genome sequences of three individuals, we investigate the genome composition with respect to a mixture between *M. m. domesticus* and *M. m. musculus*. Further, we study also morphological patterns of the mandible based on contemporary and historic samples. We conclude that *M. m. helgolandicus* constitutes indeed a separable genetic unit with specific adaptations that effectively lead to a reproductive isolation, even in the face of immigrating mice from the mainland. Their designation as subspecies appears therefore justified.

## Results

### Molecular assignment of *M. m. helgolandicus*

For an initial molecular assignment of the ancestry of *M. m. helgolandicus*, we used four nuclear diagnostic markers to relate them either to *M. m. musculus* or *M. m. domesticus* (Additional file 1: Table S1)*.* For all four markers, including one for the X-chromosome and one for the Y-chromosome, we found only the *M. m. domesticus* variants among all Heligoland mice*.* This supports the previous classifications [12–16] and the notion of colonization from neighboring Northern German or Danish populations, which are *M. m. domesticus* [16].

**Fig. 2:**
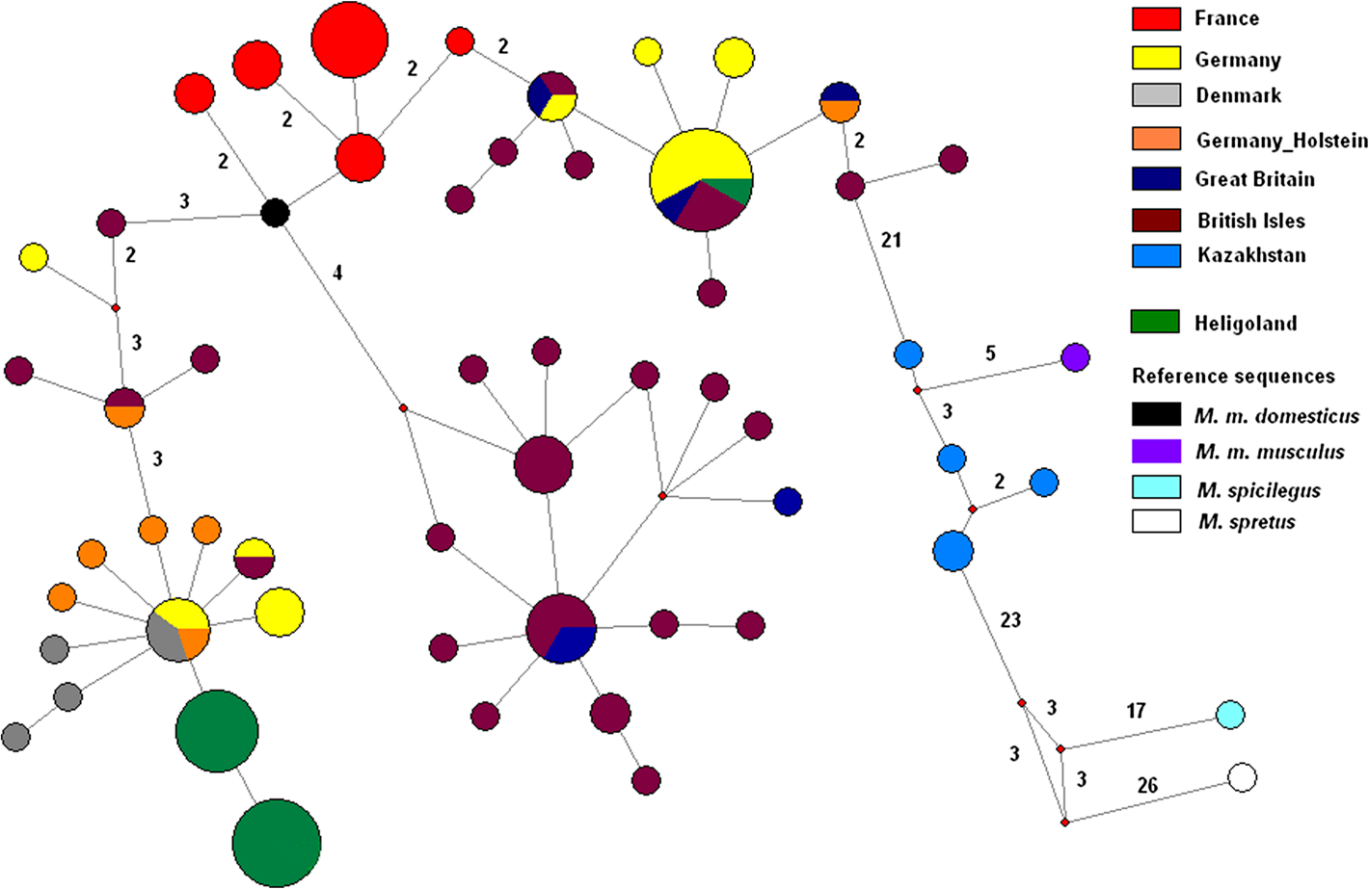
mtDNA D-loop haplotype network. Network based on mtDNA D-loop haplotypes calculated using Median Joining. Sequences include the haplotypes of the mice caught on Heligoland as well as previously published sequences *M. m. domesticus* and *M. m. musculus* populations and *Mus* reference sequences. The area of each circle is proportional to haplotype frequency. Each node is one mutational step away from the next one (excluding insertions/deletions); numbers indicate the cases of more than one step. Small red circles indicate branch splits

Mitochondrial D-loop sequencing revealed only two major haplotypes that are both unique to Heligoland whereby one is derived from the other by a single mutational step (Fig. 2). It is most closely related to a haplotype occurring in Denmark and Germany. We also found an insertion of 11 bp in all the individuals representing these two haplotypes. This insertion was previously found in one sequence from Heligoland and was named as a distinct haplotype DEU_U47469.41 Holstein [16], although the same insertion was also found in a few mainland populations, i.e. it is not diagnostic for the Heligoland haplotype.

Hence there is nowadays only a single colonizing haplotype left on the island, either because only one was associated with the colonizing mice, or other variants got lost by drift. The one remaining haplotype has further evolved on the island and no further introgression by other haplotypes seems to have occurred.

However, there is one exception to this pattern. We found a single individual with a very different haplotype that is otherwise known from Germany and Britain (Fig. 2). As further detailed below, this individual differs also in all other respects from the Heligoland mice and we interpret it as a recent immigrant (see Discussion).

The microsatellite analysis showed low levels of genetic diversity, i.e. reduced heterozygosity and only few alleles for Heligoland mice (Table 1). This is likely due to a combination of a colonization bottleneck and the small effective population size on the island.

**Table 1:** Population genetic parameters of the *M. m. helgolandicus,* various *M. m. domesticus* and one *M. m. musculus* (Kazakhstan) populations for microsatellite loci typed in this study

Location	Population	N	H_obs_	H_exp_	A_av_
Heligoland	Heligoland	17	0.33	0.48	3.3
Germany	Cologne-Bonn	45	0.53	0.80	11.2
	Plön-District	18	0.38	0.77	7.9
	Schömberg	12	0.44	0.70	6.5
France	Massif Central	46	0.60	0.77	11.0
	Louan	12	0.55	0.73	5.8
	Divonne les Bains	12	0.59	0.79	7.8
	Nancy	12	0.60	0.80	7.2
Kazakhstan	Almaty	47	0.61	0.76	13.2

*N* number of individuals scored, *H*_*obs*_ observed heterozygosity, *H*_*exp*_ expected heterozygosity, *A*_*av*_ mean number of alleles per locus

**Fig. 3:**
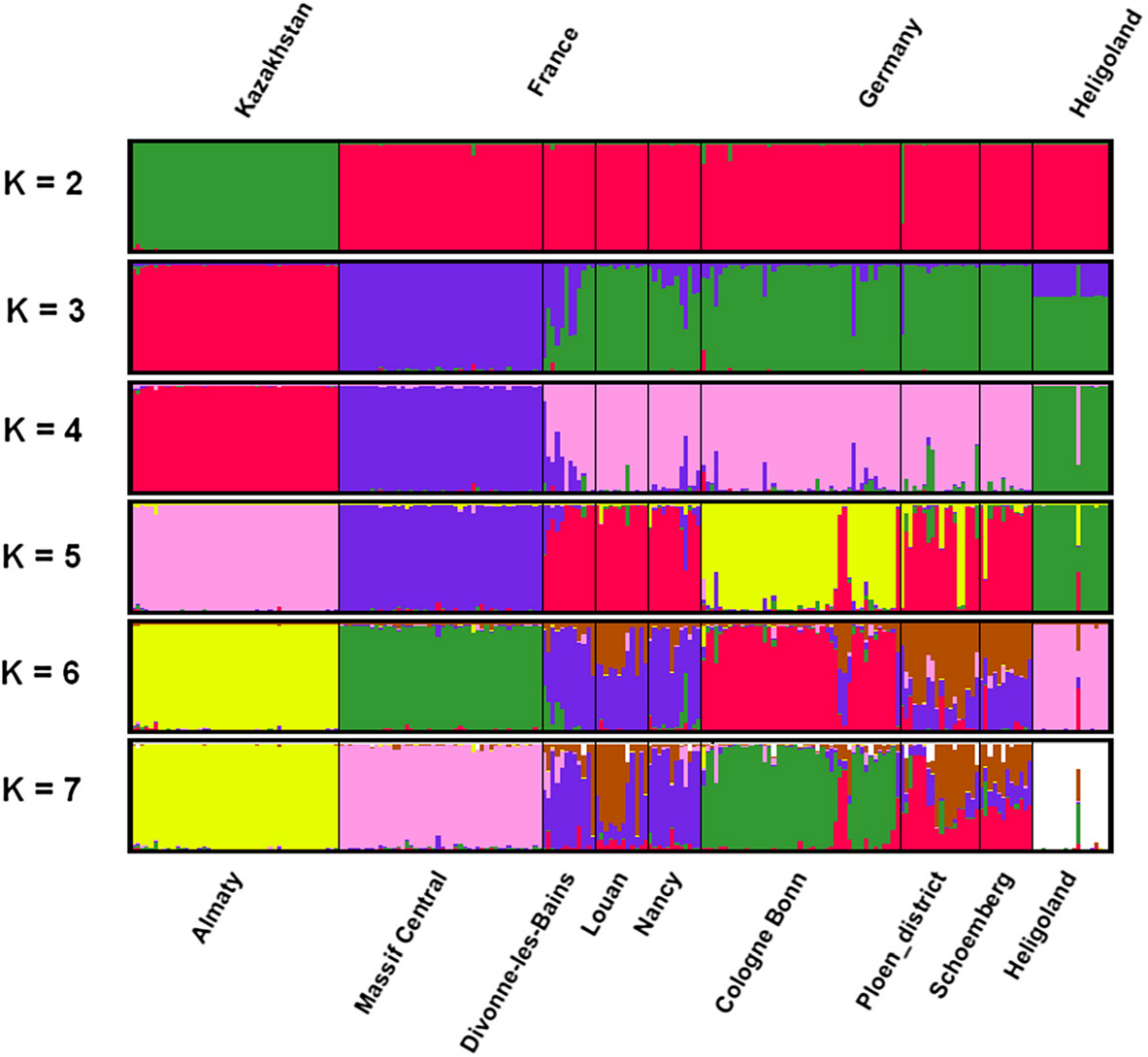
Population analysis based on STRUCTURE. Clustering of 221 individuals from 9 *Mus musculus* populations assuming K = 2-7 clusters. The optimal number of clusters is two, the mean (across replicate runs) log likelihood for K = 2 was (-18374.19). Each individual is represented by a column divided into K colors with each color representing a cluster. Different populations are separated by a black line and are labeled below the figure by sample locations and above the figure by geographic region

Microsatellite based STRUCTURE analysis assigned the Heligoland individuals to *M. m. domesticus* at K = 2 and to a distinct population at K values > 3 (Fig. 3). Only the presumed immigrant mouse shows a different assignment and associates with populations from mainland Germany.

### Colonization of the island

To obtain a time estimate for the initial colonization of the island, we made use of a calibration obtained for the colonization of Kerguelen Islands which is based on full mitochondrial sequence data [17]. We had previously shown that the occurrence of new mutations in mitochondrial DNA of newly colonized islands reflects mostly the primary mutation rate, i.e. includes mutations that would be removed by purifying selection at later stages of evolution [17]. This observation provides a solid basis for dating very recent immigration events for mice, at least with respect to their mitochondrial lineage.

We sequenced 11 full mitochondrial genomes of Heligoland mice carrying the colonizing haplotypes. We found a total of 10 positions with new point mutations (Table 2). This does not include the mutations in the D-loop, to make the frequency calculations comparable with the ones in ref [17]. The 10 mutations correspond to a frequency of 5.9 x 10^-5^ per nucleotide sequenced. Three of the mutations occur in otherwise highly conserved regions (pos. 5157 in tRNA-Asn, pos. 5163 in the replication origin and pos. 15,163 in the cytb gene, causing an amino acid-change from Gly to Ser) (Table 2). This confirms the notion that potentially slightly deleterious mutations can segregate in the population for some time after a new colonization [17].

**Table 2:** Mitochondrial genome mutations (except in control region) found in the mice on Heligoland. The positions refer to the genome reference sequence (NCBI37/mm9), the consensus sequence is the one found for the haplotypes on Heligoland

	tRNA^val^	ND2	tRNA^Asn^	rep_ori	ND4	ND4	ND5	ND6	CYTB	CYTB
Position	1080	4771	5157	5163	10688	10689	12009	13681	14698	15163
Consensus sequence	A	C	C	C	T	A	T	C	T	G
HG_01		T								
HG_02								A		
HG_05							C			
HG_06										A
HG_08	G									
HG_10										
HG_11										A/G^a^
HG_12									C	
HG_13									C/T^a^	
HG_14					C	T				
HG_1450_2			T	T						
Rat	A	C	C	C	C	A	C	A	T	G
Human	A	C	C	C	A	A	A	C	T	G
Orangutan	A	C	C	C	T	A	C	C	T	G
Dog	T	T	C	C	A	A	G	G	T	G
Horse	A	A	C	C	A	A	G	A	T	G
Opossum	T	A	C	C	A	C	A	C	T	G

^a^Heteroplasmic positions in the respective animals inferred from double peaks in the sequence reads - not counted as new mutations, since they are also present in another animal in the study, which suggests inheritance

In the Kerguelen mice we had found a mutation frequency of 3.0 x 10^-5^ per nucleotide and these islands were colonized about 200 years ago, based on historical records (arrival of the first ships from Europe) [7]. This suggests that the mice on Heligoland are approximately two times as old as those on Kerguelen, i.e. the mice carrying the colonizing haplotype would have arrived around 400 years ago, at least when one assumes that they have comparable generation times (see [17] for discussion of this point).

### Admixture with *M. m. musculus* genomic sequences

**Fig. 4:**
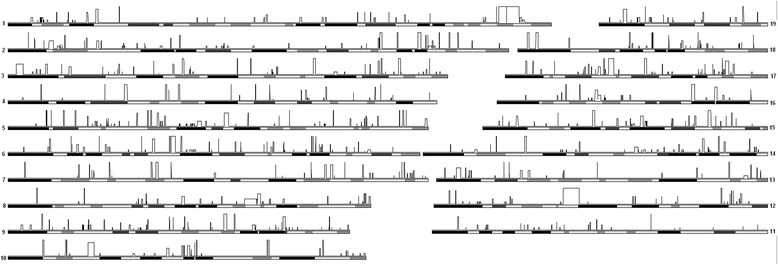
Genome introgression into *M. m. helgolandicus* from *M. m. musculus*. Patterns of introgression into the autosomes of *M. m. helgolandicus* from Heligoland visualized with the Genome Graphs utility of the UCSC Genome Browser [54]. The size of the bars represents the number of haplotypes of *M. m. musculus* found at the respective position among the three animals analysed

The morphologic descriptions by Zimmermann [12] and Reichstein and Vauk [13] had suggested a mosaic of characters between *M. m. domesticus* and *M. m. musculus*. Although the small subset of diagnostic molecular markers tested above had assigned all Heligoland mice to *M. m. domesticus*, we were interested to assess by how far admixture by *M. m. musculus* genomic sequences might have contributed to the genomic makeup of the Heligoland mice. Such an admixture is also known for mice on the Faroe Islands [18] and given the proximity of the Danish and Northern German mice to the hybrid zone with *M. m. musculus* [16], it would seem possible that the colonizing mice have carried some *M. m. musculus* alleles. To obtain a genome-wide estimate on possible admixture, we have sequenced the full genomes of three Heligoland individuals at an approximately 11x coverage. As a reference panel to assess admixture, we used the high density single nucleotide polymorphism (SNP) data obtained for *M. m. domesticus* and *M. m. musculus* populations in [19]. We extracted the respective SNP positions from the Heligoland genome reads and used Hapmix to assign Heligoland genome blocks to either *M. m. domesticus* or *M. m. musculus*. We find that about 6.5 % of the genome shows at least partial admixture across the autosomes, i.e. at least one *M. m. musculus* haplotype was predicted to occur in the Heligoland mice (Fig. 4; Additional file 2: Table S2). This is higher than the average frequencies we found in *M. m. domesticus* populations distant from the hybrid zone (2.8 - 4.7 % in Western Germany and France) but lower than for a *M. m. musculus* population close to the hybrid zone (17.7 % in the Czech Republic) [19]. On the other hand, since only three individuals were analyzed for Heligoland versus eleven from the mainland populations [19], the percentage of admixture with low frequency *M. m. musculus* haplotypes could be higher in Heligoland as well. Overall, the pattern of admixture is compatible with the assumption that colonizing mice have originated from an area close to the hybrid zone and may have carried *M. m. musculus* genetic material upon colonization of the island.

Given the mosaic nature of the phenotype of the Heligoland mice [13, 14], the *M. m. musculus* alleles could have contributed to the island-specific characteristics and adaptations. If this was the case, they would be expected to have become fixed in the Heligoland mice. To assess this, we have looked specifically at the genome fraction that is apparently fixed for *M. m. musculus* haplotypes (i.e. 6 copies in the three animals). Gene ontology (GO) analysis of these genome blocks suggests essentially two distinct functional terms, namely an enrichment of genes involved in sensory perception of smell and regulation of different responses in this fixed genome fraction (Additional file 3: Table S3). However, upon closer examination of the fixed *M. m. musculus* blocks, this is mostly due to an about 2 MB genome region on chromosome 17qB1 covering part of an olfactory receptor gene cluster and an about 1 MB genome region on chromosome 1qB1 covering part of an interleukin 1 receptor-like 2 precursor gene cluster (see Discussion).

### Mandible shapes

**Fig. 5:**
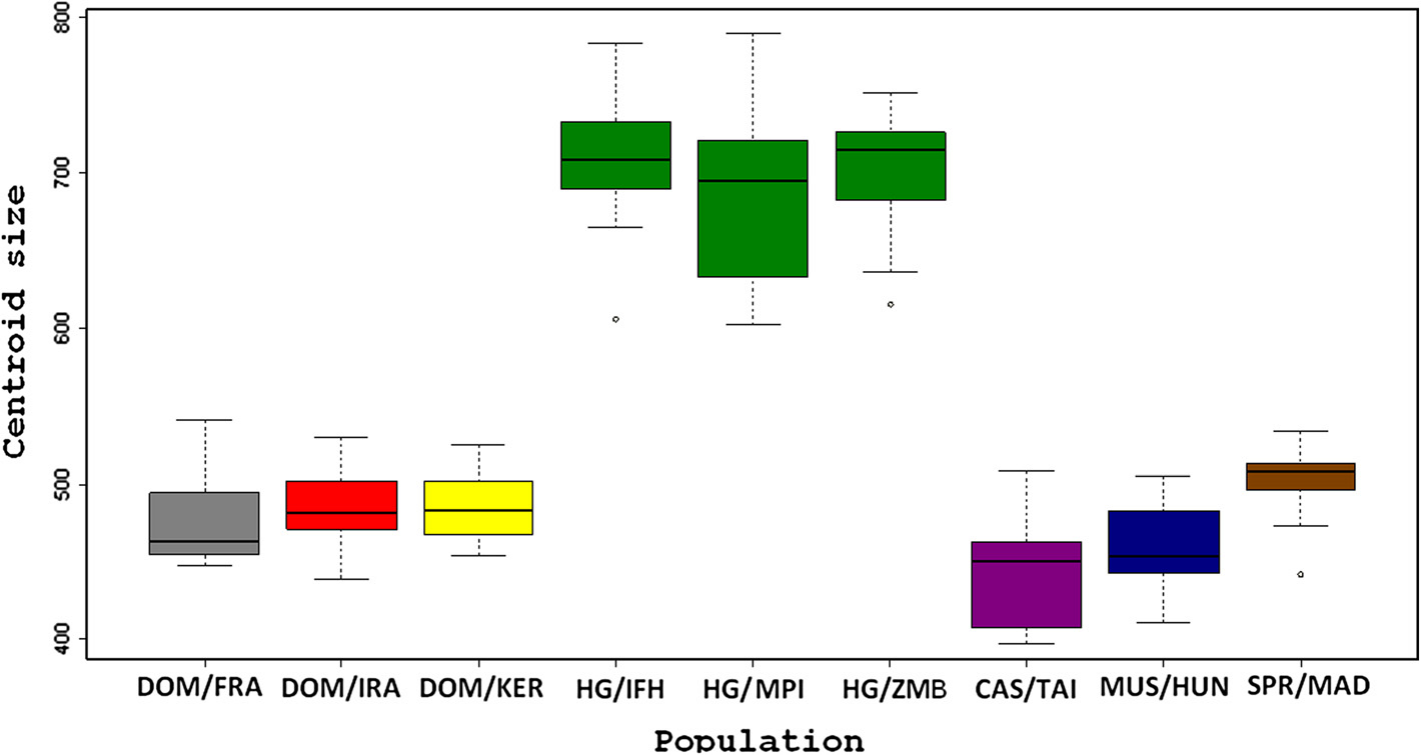
Box plot of the mandible centroid size. Box plot of centroid size in populations of the house mouse from Heligoland and mainland species. The house mice from Heligoland are represented by three different collections are shown in dark green color (HG/ZMB from the 1930s, HG/IFH from 1952-1970, HG/MPI from 2004-2012). *M. m. domesticus* is represented by a population from Frankfurt (DOM/FRA in grey), from Ahvaz, Iran (DOM/IRA in red) and Kerguelen Islands (DOM/KER in yellow). *M. m. castaneus* is represented by a population from Taiwan (CAS/TAI in violet), *M. m. musculus* by a population from Hungary (MUS/HUN in blue) and *M. spretus* by a population from Madrid (SPR/MAD in brown). Averages and inter-quartile ranges are shown, outliers are indicated by small circles

**Fig. 6:**
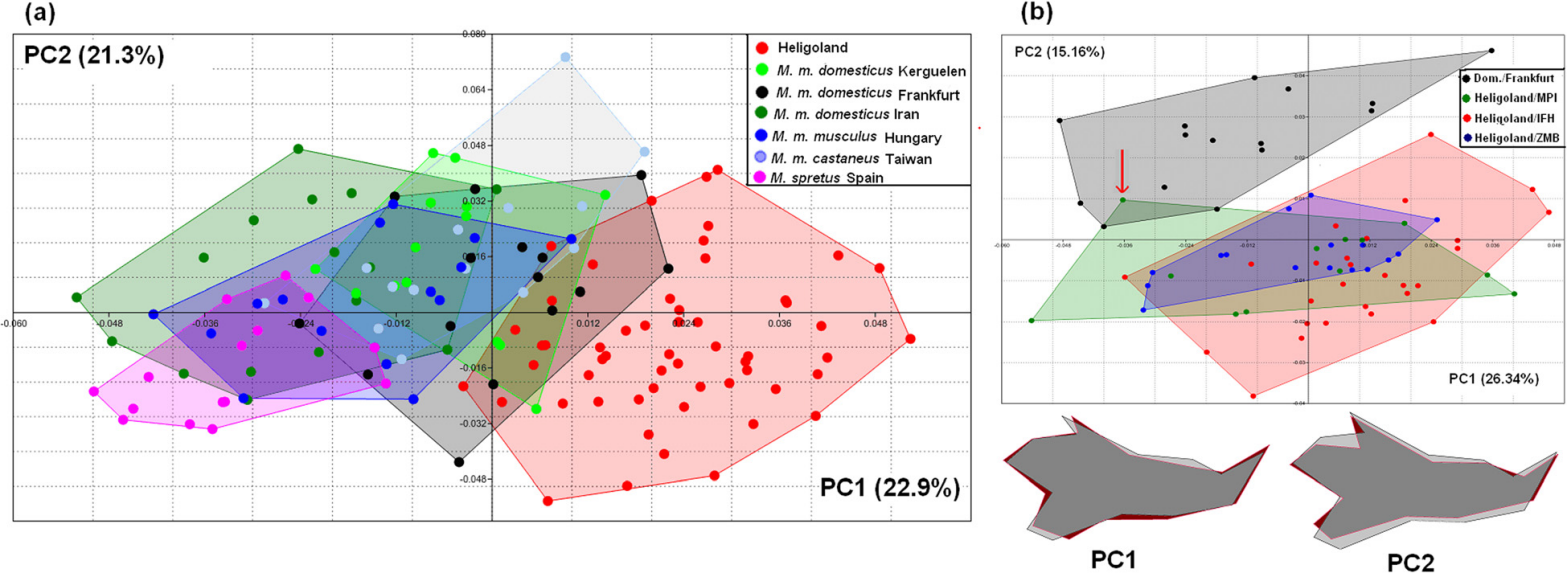
PCA analysis of mandible shape between *M. m. helgolandicus* and reference populations. **a** General comparison with multiple populations. The first two axes of a PCA scatter plot are shown. **b** Comparison of each time sample from Heligoland with the reference population from Germany. The first two axes of a PCA scatter plot are shown on top; the shape changes along the first two PCs between *M. m. helgolandicus* and *M. m. domesticus* populations are depicted as wire-frame graphs at the bottom. Shape changes are from grey (*M. m. domesticus*) to red (*M. m. helgolandicus*). The red arrow points to the individual that represents a recent immigrant (see text)

Geometric morphometrics allows a detailed characterization of shape differences between taxa [20] and the mouse mandible has been used to generate a large reference data set for populations and sub-species of *Mus musculus* [21]. We applied here geometric morphometrics and principal component analysis to three sets of skull samples from Heligoland, collected at different time points. The first one is from the 1930s and represents the pre-war sample that was also used by Zimmermann to describe the subspecies (i.e. the type material). The second was collected in the years after the re-population of the island up to the 1970s and the third is from our recent own collection (2004-2012). We find that the mandible of *M. m. helgolandicus* differs from the reference populations, both with respect to size (Fig. 5) and shape (Fig. 6), but not very much between the sampling periods.

Centroid sizes of all three Heligoland samples are significantly larger (*P* < 0.0001) than the mainland species, including *M. m. domesticus*, *M. m. musculus*, *M. m. castaneus* and *M. spretus* (Fig. 5). While it is known that mice can become larger after colonization of islands [22, 23], the centroid size is not a direct measure of absolute size but depends also on anisotropic differences of landmark positions between samples [24]. In fact, the overall size (as determined by simple linear length measurements) and weight of *M. m. helgolandicus* specimens was not found to be larger than the mice of the neighboring mainland populations [13]. Interestingly, a centroid size increase is not evident for the mice on the Kerguelen Islands (Fig. 5), i.e. this points to a rather specific size and shape adaptation of *M. m. helgolandicus* mandibles. The three samples form the three different time points do not show significant centroid size differences, i.e. the change must have happened early during the colonization process. A similar rapid adaptive shift after colonization of an island was shown for tooth shape changes of voles on the Orkney Islands [25].

The overall shape analysis via principal component analysis (PCA) shows that the Heligoland mice are also distinct from individuals of *M. m. domesticus* populations, as well as the other subspecies (Fig. 6a). A small overlap is only seen with the German and the Kerguelen *M. m. domesticus* populations in the overall analysis (Fig. 6a). To assess in more detail whether any major shape changes are evident between the three time samples from Heligoland, we have compared these with the German population. We find that the three time samples overlap very much with each other but very little with the German population (Fig. 6b). In fact, the one animal overlapping with the shape space of the German population (arrow in Fig. 6b) is the one that is suspected to be a recent immigrant based on the molecular analysis (see above). Hence, the consistent distinction of Heligoland mice across the three time-samples suggests that not only the size, but also the shape evolution has occurred early during the colonization phase and is not further ongoing.

The outline of the shape changes relating to PC1 and PC2 with respect to the German reference population are depicted at the bottom of Fig. 6b. They are characterized by a general elongation of the mandible as well as sharper angular and condylar processes. These characteristics point towards an adaptation to a carnivorous/insectivorous diet [26, 27] rather than the omnivorous diet that is otherwise typical for the genus *Mus*. A similar diet shift from plant seed to macro-invertebrates has been documented for mice on sub-Antarctic islands [28]. However, the mandible shapes between Kerguelen and Heligoland islands are nonetheless distinct from each other (Fig. 6a).

## Discussion

The results of our study confirm the notion that the house mice on Heligoland constitute a distinct lineage, both molecularly and morphologically. Most interestingly, although the separation from the mainland populations may be relatively recent, the now established population seems to be very refractory to further genetic immigration. This finding is compatible to that from Kerguelen archipelago and Sub-Antarctic Islands, where we found that when a population is established and settled on a small island, further introductions are not effective to visibly interfere with the genetic composition of the resident population [7]. In the case of Heligoland it is particularly interesting to see that this resilience against re-invasion has been maintained in spite of major historical disturbance and a likely high influx of new mice from the mainland via ships. Heligoland belonged to England during most of the 19th century, but we find no indication of haplotypes from England on Heligoland. Similarly, the heavy use of Heligoland as military harbor during the 20th century, with large numbers of provisioning ships coming in did not leave signs of immigration of mice from the mainland behind.

Shortly after World War II, the island suffered the complete evacuation of the human population, as well as heavy bombing and explosions. Rebuilding the village after this period would have again brought in a large amount of material and provisioning and this is ongoing until today, since the island has a tax-free status with large numbers of day-time visitors coming for shopping. In spite of this, we could identify only a single individual that did not fit into the pattern of Heligoland mice. Given the small size of the island, it is very unlikely that this individual represents a separate and otherwise undetected sub-population on the island. It is much more likely that it has come in recently by a ship and thus supports the notion that there is indeed an immigration pressure through incoming mice.

### Initial colonization

The molecular dating based on counting the new mutations in the colonizing mitochondrial haplotype suggests an initial colonization about 400 years ago. However, there is considerable uncertainty associated with this estimate, since it depends on the assumption of a linear accumulation of new mutations during this time frame. Both, coalescence processes due to limited population size, as well as selective removal of slightly deleterious mutations would lead to an underestimate of the colonization time. Hence, a somewhat older colonization is also possible. Another possibility that would extend the estimate for the initial colonization time would be that a first colonizing haplotype was lost and replaced by a later incoming one. However, we consider this less likely, given the general pattern of resilience against re-invasion of new mitochondrial haplotypes.

Settlements in Heligoland are already known from the Neolithic and the Bronze Age, but *M. m. domesticus* arrived only less than 3000 years ago in Western Europe, i.e. these early settlements could not have led to the establishment of mouse populations on Heligoland. Friesian settlements on Heligoland are known since the Middle Ages (7th century), but the molecular dating above is not very well compatible with such an early mouse colonization, even if one takes an extended time scale into account. On the other hand, it would be entirely possible that there was a first colonization with house mice around this time, but followed by an extinction, as it was suggested in the case of the colonization of the Canary islands [29]. The currently existing mice are most likely derived from a colonization at some point between the 12th-18th century where Heligoland belonged first to the Kingdom of Denmark and later to the Duchy of Schleswig Gottorp, During this time it was economically active in processing copper ore with corresponding regular ship traffic. A colonization during this time would also explain the molecular proximity of the Heligoland mitochondrial haplotype with the ones from Northern Germany and Denmark.

### Insular adaptation and genomic introgression

The distinct slender shape change of the mandible compared to mainland populations provides a hint for a specific adaptation, namely a trend towards carnivory [26, 27]. It seems possible that mice on Heligoland have started to use worms, insects and bird carcasses in their diet, as it was also observed for Kerguelen mice [28]. While diet could also have a plastic influence on shape components of the mandible, comparative experiments with extreme diet differences showed that the plastic influence is only moderate [21] and would not be sufficient to explain the specific shape shift seen in the Heligoland mice. On the other hand the relatively broad scatter of shape in the PCA analysis (Fig. 6) in spite of a relatively homogeneous genetic background of the island mice could imply a larger role for environmental influence on shape than for the mainland populations.

The introgression of genomic regions from *M. m. musculus* may have contributed to the seemingly mosaic phenotype [12, 13] and other island-specific adaptations. Although a pattern of introgression of genomic regions between the subspecies is also known for mainland populations [19], the relative amount on *M. m. musculus* genomic material in *M. m. helgolandicus* appears to be somewhat larger than we would have expected to find in *M. m. domesticus* populations, in particular when one takes into account that only three individuals were analyzed so far. However, the Danish and Northern German populations of *M. m. domesticus* are close to the hybrid zone with *M. m. musculus* [16], implying that already the colonizing mice may have harbored an increased amount of *M. m. musculus* genetic material compared to other mainland populations. Alternatively, there may have been some small amount of gene flow of *M. m. musculus* haplotypes after the initial colonization, but there is no obvious shipping route that would connect Heligoland to areas of *M. m. musculus* prevalence. It is also unlikely that *M. m. musculus* introgression would have occurred after the Heligoland population stabilized, given that it shows this strong resilience even towards *M. m. domesticus* introgression.

Given the increasing realization that genomic admixture can be a creative force in evolution [30, 31], one may speculate that the *M. m. musculus* genetic material could have been involved in some island specific adaptations. However, with the uncertainty about the true source population for the Heligoland mice, this would be very difficult to ascertain. Although the GO analysis of *M. m. musculus* fixed regions showed an overrepresentation of some gene classes, this does not necessarily imply adaptation. Given that random fixation of blocks including gene clusters would also result in a respective GO-term enrichment, it is not possible to distinguish whether the enrichment has occurred by adaptation or random fixation. Accordingly, although the mosaic phenotype could have suggested an adaptive contribution of *M. m. musculus* genetic material, we have currently no way to unequivocally pinpoint the parts of the genome that could have been responsible for this.

## Conclusion

Our data show that the mice on Heligoland have developed a specific adaptation to the island conditions and have maintained their genetic and morphological identity, i.e. they are true “Helgoländer”. We propose that this justifies their designation as separate subspecies, although they have split only very recently from the mainland populations. Given this very recent separation, they have of course not the same rank as the other well recognized subspecies (*M. m. domesticus*, *M. m. musculus* and *M. m. castaneus*). But since that the name *M. m. helgolandicus* is already established, we recommend to retain it.

## Materials and methods

### Ethics statement

Mice were maintained and handled in accordance to Federation of European Laboratory Animal Science Association (FELASA) guidelines and German animal welfare law (Tierschutzgesetz § 11, permit from Veterinäramt Kreis Plön: 1401-144/PLÖ-004697).

### Sample collection

A total of 17 mouse individuals from Heligoland Island were collected in the period 2004-2012 by researchers at the Institute for Avian Research and by ourselves in summer 2012. The 2012 collection was done in a single field trip from two localities known as upper and lower lands. Mice were trapped live and for each mouse body weight, body measurements and coat color were scored for dorsal and ventral parts using Turner’s standard color chart (Additional file 4: Table S4). The mice were sacrificed by CO_2_ inhalation and were dissected on site and organ tissues from each mouse were prepared and later each mouse was preserved in absolute Ethanol for morphological analysis and future analysis.

### DNA extraction

Total genomic DNA was extracted from mice tissue samples (mostly liver) using a salt extraction protocol. The tissue was incubated in a lysis buffer (80 mM EDTA, 100 mM Tris, 0.5 % SDS) with Proteinase K (0.20 mg/mL) at 55 °C overnight on a slowly shaking platform. 500 μL 4.5 M sodium chloride was added to precipitate fat and proteins. Then 300 μL chloroform was added to separate the DNA from the protein and lipid phase. DNA was precipitated using (0.7 of the total volume) pure Isopropanol and the DNA pellet was washed with 500 μL 70 % Ethanol and dissolved in 30 μL Tris-EDTA (TE) buffer (10 mM Tris, 0.1 mM EDTA, pH 8.0).

### Diagnostic nuclear markers

Extracted genomic DNA was used to analyze four nuclear genetic markers that are known to differentiate between *M. m. domesticus* and *M. m. musculus*. Specific primers for each genetic marker were used and each sample was amplified by polymerase chain reaction (PCR) and scored by gel electrophoresis. The Androgen binding protein (*Abp)* marker was tested for PCR subspecies-specific alleles as in [32, 33]. *D11 cenB2*, a marker at the centromeric region of chromosome 11 was typed for PCR subspecies-specific alleles as in [34]. Bruton agammaglobulinemia tyrosine kinase (*Btk)* marker found on chromosome X was typed and scored as in [35] for the presence or absence of the B1 insertion in the *Btk* gene. The Zinc finger protein 2, Y chromosome linked (*Zfy2)* marker was tested for the absence or presence of an 18 bp deletion following the protocol of [35]. The details of the primers used are listed in Additional file 1: Table S1.

### Microsatellite typing

We chose 21 microsatellites (details provided in Additional file 5: Table S5) from [36] to genotype the populations from Heligoland with populations from France and Germany collected by [37, 38] and a population from Northern Germany (district of Plön) collected by our colleagues at the Institute in 2007. Of each primer set the forward primer was labeled with FAM or HEX dye on the 5' end. The PCR reactions were carried out in 5 μL final volumes using 5 ng DNA template and the standard protocols of QIAGEN Multiplex PCR kit. The PCR was programmed as follows: initial incubation step at 95 °C for 15 min followed by 28 cycles at 95 ° C for 30 s, 60 °C for 1.30 min, 72 °C for 1.30 min with a final extension at 72 °C for 10 min. PCR products were diluted 1:20 in water. 1 μL of the diluted PCR product was added to a previously prepared mixture of 10 μL HiDi formamide and 0.1 μL of 500 ROX size standard (Applied Biosystems, USA). A denaturation step was then performed with the following incubation times: 90 °C for 2 min and 20 °C for 5 min. The samples were analyzed using GeneMapper version 4.0 for Windows (Applied Biosystems, USA).

The genotyped data from this study were combined with data for three populations from Kazakhstan, Germany and France, genotyped previously for the same microsatellite loci [36, 37]. The total number of individuals analyzed was 221 from a total of 9 populations. The number of individuals per population and their geographical locations are detailed in Table 1. The average number of microsatellite alleles per locus and the observed and expected heterozygosities were calculated per population using the POPGENE program version 1.32 [39]. To depict the population structure among the populations, we used the software STRUCTURE version 2.3.3 [40, 41]. Of each independent run we employed the admixture model for individual ancestry and the F model for allele frequency correlation and without prior information on localities of samples. We used 1,000,000 Markov Chain Monte Carlo (MCMC)) repetitions and a burn in of 100,000 iterations with a number of clusters K from 1 to 12, each simulated ten times.

### mtDNA sequencing

The mtDNA D-loop was amplified using the primers 5'CATTACTCTGGTCTTGTAAACC and 5'GCCAGGACCAAACCTTTGTGT from [7]. The reactions were carried out in 10 μL final volume with the following cycling parameters: 95 °C for 15 min followed by 35 cycles of 95 °C for 30s, 60 °C for 1.30 min, 72 °C for 1 min and an elongation step at 70 °C for 15 min. Samples were then purified with Exonuclease/Shrimp Alkaline Phosphate (Exo/SAP) (USB Corp.) with the following incubation conditions: 37 ° C for 20 min and 80 ° C for 20 min. Then each of the amplified sequences was subjected to a cycle sequencing reaction using the following conditions: 96 °C for 1 min followed by 29 cycles of 96 °C for 10 s, 55 °C for 15 s and 60 °C for 4 min. The sequences were edited and visualized using CodonCode Aligner version 4.1.1 (CodonCode Corp.) and were aligned with previously published data obtained from [8, 16, 37] using MEGA version 5.0 [42]. The network was calculated using the Median Joining method and drawn with Network version 4.5.1.0 (Fluxus Technology Ltd.) [43]. Mitochondrial genomes were sequenced for 9 mice using a set of primers described in [17, 44] and provided in Additional file 6: Table S6. The sequences were edited and visualized using CodonCode Aligner version 4.1.1 (CodonCode Corp.). Three mitochondrial genome sequences were additionally obtained from the whole genome sequenced data (detailed below). A total of 12 mtDNA genome sequences were aligned using MEGA version 5.0 [42]. We determined the number of mutations in these sequences in comparison to the consensus sequence and we estimated the mutation frequencies from the total number of nucleotides sequenced using the procedure applied by [17, 44]. One of the genomes was derived from the mouse that had recently immigrated - this was not used for mutation frequency statistic. The sequences were submitted to Genbank and are available under accession numbers KP877610 to KP877620.

### DNA library construction and genome sequencing

DNA library preparation was carried out by the sequencing center according to the standard Illumina TruSeq protocol for sequencing on HiSeq 2000 (Illumina Inc., San Diego, USA). Consequently, two paired-end libraries with insert size of ~230 bp were generated for deep sequencing of each genome using HiSeq 2000 (Illumina Inc.). The constructed DNA libraries for the 3 samples were tagged and then pooled and sequenced with a paired end cluster generation kit on 6 Illumina HiSeq2000 (2x100bp) lanes, resulting in 70-80Gb of filtered data for each sample. The raw sequence reads were deposited in the European Nucleotide Archive (ENA; http://www.ebi.ac.uk/ena/) under project accession number PRJEB9450 and sample accession numbers SAMEA3416740 to SAMEA3416742 . The paired-end reads obtained from the previous step in FASTQ format were subjected to a trimming step using Trimmomatic version 0.30 [45]. The trimming step consists of trimming low quality bases and removal of adapters and other Illumina-specific sequences and dropping of reads below 60 bases long [45]. Paired end reads were mapped to the indexed mouse reference genome (NCBI build 37/mm9) [46] by sequence alignment (aln) using the Burrows Wheeler Aligner (bwa) version 0.6.2-r126 [47]. The mapped reads were produced in Sequence Alignment/Map format (SAM) [48] and were subjected to Samtools utility functions view, sort and index respectively to produce the Binary sequence Alignment/Map format (BAM). PCR duplicates were removed using the rmdup function provided by Samtools utility. The mpileup function of samtools version 0.1.18 was used to detect single nucleotide polymorphisms (SNPs) with respect to the reference genome (NCBI build 37/mm9) [49] along with the bcftools view function version 0.1.17-dev [49]. The vcftools version 0.1.9.0 was used to generate the variant call format file which is a representation of the respective sequence variations of the analyzed sequences [50].

### Inference of local ancestry in admixed populations

To characterize patterns of introgression across the genomes of the three house mice from Heligoland, the hidden Markov model approach implemented in Hapmix software was used. Hapmix [51] is used mainly to infer the ancestral state of a given admixed individual for all possible chromosomal segments in respect to two hypothetical potential source populations. Hapmix treats the two hypothesized source populations as totally phased and combines a phasing algorithm that allows the calculation of the average inferences about ancestry over all the possible phased haplotypes. Hence, it compares the unphased data from putatively admixed individuals to the phased data from the reference ancestral populations [51]. The phased data for the reference populations are based on SNP-microarray data (mouse genome diversity array [52]) and were obtained from [19]. Each reference population was represented by 22 autosomal chromosome samples from 11 unrelated wild caught individuals [19].

Given that, the mouse genome diversity array was annotated according to the mouse dbSNP128, we used the functional annotation of genetic variants implemented in ANNOVAR [53] combined with the dbSNP128. The annotated variants from our genomes were used to detect overlapping regions with the mouse genome array data for reference populations and hence used for introgression analysis.

The patterns of introgression were depicted using Hapmix HAPLOID mode. The parameters used were 100 generations since admixture and miscopying value of 0.0005. These values have been found to detect smaller introgressed haplotypes with reasonable power. The minimum per SNP certainty threshold to call a SNP introgressed was 0.9 and the recombination parameters used as described in [19].

The haploid mode estimates the likelihood that a haplotypic region in an admixed individual from Heligoland is statistically correlated to the Kazakhstan population or to the German population studied in [19]. Introgression was explained by the inferred probabilities of an individual to have 1 or 0 copies from the first population (Kazakhstan), or 9 for unknown ancestry. Hence, if the ancestry of a chromosomal region was assigned to the *M. m. musculus* subspecies (Kazakhstan population), this region was considered introgressed. The inferred probabilities of introgression at each locus were merged with the SNP input file used for running Hapmix. The new merged file was subjected to an R script to detect the boundaries of introgressed haplotypes, their length and frequency from the number of introgressed haplotypes within a given region.

### Data visualization and GO of introgressed regions

The regions fixed for introgressed haplotypes from *M. m. musculus* were loaded as custom track on the University of California Santa Cruz (UCSC) genome browser. The Genome Graphs utility of the browser was used to visualize the genomic regions affected by introgression and to retrieve gene lists overlapping with the respective regions across chromosomes [54]. In addition, the Tables function [55] was used to calculate fractions of genome affected. Gene lists were then analyzed with the online tool GOrilla (http://cbl-gorilla.cs.technion.ac.il/) [56]. The tool was used to detect enrichment terms of genes that appear densely at the top of the ranked list of genes using *Mus musculus* reference genome. Here, we focused on ontology associated with “Biological process” with a significance threshold at P-value < 0.001 [57].

### Samples for geometric morphometrics

We analyzed a total of 65 skull specimens for the house mice from the island of Heligoland collected at different time periods (details of specimens are supplied in Additional file 7: Table S7). The oldest was collected by Zimmermann [12] early in the thirties and represents the material on which he based the name designation. It was obtained from the Zoological Museum in Berlin (ZMB) as a loan. The second was collected by amateur collectors during the 1950s-1970s and was obtained through a loan from the Institute für Haustierkunde (IFH) in Kiel. The contemporary collection at Max Planck Institute (MPI) was collected during our trip to Heligoland in 2012 and by the researchers at the Institute for Avian research in Heligoland during 2004 to 2012.

All specimens were subjected to preparation prior to the scanning process following the same protocol. The skulls from the contemporary collection were prepared from whole body specimens (preserved in Ethanol) by first decapitating the head in a process that ensured that the whole skull with the mandible attached were complete. The old material borrowed from the Museum and the Institute für Haustierkunde (IFH) in Kiel were prepared taking care that the mandible remained intact and attached to the skull, for these specimens we used the provided information for sex and labeling from the containers of the borrowed material. In some cases mandibles were only available without skulls or in only one intact hemimandible. The 65 skull specimens were scanned with a micro-computer-tomograph (microCT- VivaCT 40, Scanco, Bruettisellen, Switzerland). The left hemimandible of each of the specimens scanned was outlined using the software options provided by the microCT.

### Mandible landmarking

Two dimensional coordinates of 14 mandibular landmarks were digitized on each hemimandible of the scanned and outlined specimens. In addition, incomplete mandibles due to damage resulted from snap trapping of mice or the impact of museum storage processes, were digitized by either using the intact hemimandible (left/right) or the best available landmarks. The digitization was performed in two independent rounds to reduce technical errors. The digitization was performed using two software utilities from Morphometrics tpsUtil [58] and tpsDig [59] respectively. The positions of the landmarks analyzed here were gleaned from [21].

To avoid the observer factor in landmark assignment, we selected randomly 14-16 specimens (hemimandible) from a number of populations studied by [21] and digitized them all for a combined analysis with collections from Heligoland by the same person (H.B.) who had done the digitizing for the samples described above (Additional file 8: Table S8). The subspecies *M. m. domesticus* is represented by three different populations from Germany (Frankfurt), Iran (Teheran) and Kerguelen (Gouillou). The subspecies *M. m. musculus* is represented by a population from Hungary. A population from Johnston Atoll in Taiwan was included to represent *M. m. castaneus*. And a population from Madrid was also included as a representative for the species *M. spretus*.

To avoid distortion of statistical analysis a few samples of each data set were excluded, either for the suspected young age or for the suspected old age as well as mandibles with malformation diagnosis.

### Geometric morphometrics analysis

The landmark coordinates for the different data subsets were processed with the Procrustes fit implemented in MorphoJ [60]. MorphoJ implements a full Procrustes superimposition method and is performed to produce new variables for the analyzed mandible shapes which corresponds to the raw coordinates. The superimposition translates the configurations of the raw coordinates to a point where only the shape between landmarks is the major differentiating factor [61]. The landmark coordinates derived from application of Procrustes fit in MorphoJ were then used to generate one covariance matrix for the dataset from Heligoland and another for the whole data set.

The size of the mandible for each specimen was estimated from its calculated centroid size in MorphoJ. The centroid size of the mandible is calculated as the mean values of 3 coordinates (x, y, z) for all the 14 landmarks assigned. Statistically it is the square root of the sum of the squared distances between each landmark and the centroid of the mandible and it is proportional to the square root of the mean of all squared landmark distances. It is not a direct measure of the size, simply because it is calculated for different configurations of landmarks used to summarize the shape [24]. Centroid size was calculated mainly to test for differences in size among populations and they were visualized using box plots.

The Covariance matrices obtained from the datasets were used to inspect mandible shape differentiation among and within populations from Heligoland and the mainland. The differentiation was first assigned using the multivariate analysis implemented in PCA. PCA is a widely used method for exploratory multivariate analysis and one of its uses was applied here as an ordination method to inspect the principal features of shape variation in the dataset.

### Availability of supporting data

Nucleotide sequences for mitochondrial sequences are available at Genbank (http://www.ncbi.nlm.nih.gov/popset?DbFrom=nuccore&Cmd=Link&LinkName=nuccore_popset&IdsFromResult=808177804) under accession numbers KP877610 to KP877620. Genome sequence reads are available at European Nucleotide Archive (ENA; http://www.ebi.ac.uk/ena/data/view/PRJEB9450) under project accession number PRJEB9450 and sample accession numbers SAMEA3416740 to SAMEA3416742.

## Additional files

Additional file 1: Table S1. Details of the primers used for *M. m. domesticus* versus *M. m. musculus* assignment. (XLS 31 kb) Additional file 2: Table S2. Introgressed regions in the genome of house mouse from Heligoland and their frequencies. (XLS 88 kb) Additional file 3: Table S3. Output of GOrilla showing Gene Ontology term enrichment for the biological process in the gene list overlapping the introgressed regions into *M. m. helgolandicus*. (XLSX 11 kb) Additional file 4: Table S4. Details of weight, body and tail lengths and scored dorsal and ventral color for Heligoland house mice. (XLS 29 kb) Additional file 5: Table S5. Details of the primers used for microsatellite loci typing. (XLS 29 kb) Additional file 6: Table S6. Primers used for mtDNA genome amplification and sequencing. (XLS 37 kb) Additional file 7: Table S7. List of mice skull specimens for the mice used for this study. (XLS 36 kb) Additional file 8: Table S8. List of mandible landmarks raw coordinates. (XLS 48 kb)

## Abbreviations

mtDNA: Mitochondrial genome
SNP: Single nucleotide polymorphism
GO: Gene ontology
PCA: Principal component analysis
FELASA: Federation of European laboratory animal science association
TE: Tris EDTA
PCR: Ploymerase chain reaction
Abp: Androgen binding protein
Btk: Bruton agammaglobulinemia tyrosine kinase
Zfy2: Zink finger protein 2, Y chromosome linked
MCMC: Markov Chain Monte Carlo
Bwa: Burrows wheeler aligner
SAM: Sequence Alignment/Map format
BAM: Binary sequence Alignment/Map format
UCSC: University of California Santa Cruz
ZMB: Zoological Museum in Berlin
IFH: Institute für Haustierkünde
MPI: Max Planck Institute, Plön
